# The Efficacy of Heat Acclimatization Pre-World Cup in Female Soccer Players

**DOI:** 10.3389/fspor.2021.614370

**Published:** 2021-05-25

**Authors:** César M. P. Meylan, Kimberly Bowman, Trent Stellingwerff, Wendy A. Pethick, Joshua Trewin, Michael S. Koehle

**Affiliations:** ^1^Physical Performance Department, Canada Soccer, Ottawa, ON, Canada; ^2^Division of Sports Medicine and School of Kinesiology, University of British Columbia, Vancouver, BC, Canada; ^3^Canadian Sport Institute Pacific, Victoria, BC, Canada; ^4^Sports Performance Research Institute New Zealand, Auckland University of Technology, Auckland, New Zealand

**Keywords:** plasma volume, submaximal, football, heart rate, monitoring, training

## Abstract

The efficacy of a 14-day field-based heat acclimatization (HA) training camp in 16 international female soccer players was investigated over three phases: phase 1: 8 days moderate HA (22. 1°C); phase 2: 6 days high HA (34.5°C); and phase 3: 11 days of post-HA (18.2°C), with heart rate (HR), training load, core temp (*T*_c_), and perceptual ratings recorded throughout. The changes from baseline (day−16) in (i) plasma volume (PV), (ii) HR during a submaximal running test (HRex) and HR recovery (HRR), and (iii) pre-to-post phase 2 (days 8–13) in a 4v4 small-sided soccer game (4V4SSG) performance were assessed. Due to high variability, PV non-significantly increased by 7.4% ± 3.6% [standardized effect (SE) = 0.63; *p* = 0.130] from the start of phase 1 to the end of phase 2. Resting *T*_c_ dropped significantly [*p* < 0.001 by −0.47 ± 0.29°C (SE = −2.45)], from day 1 to day 14. Submaximal running HRR increased over phase 2 (HRR; SE = 0.53) after having decreased significantly from baseline (*p* = 0.03). While not significant (*p* > 0.05), the greatest HR improvements from baseline were delayed, occurring 11 days into phase 3 (HRex, SE = −0.42; HRR, SE = 0.37). The 4v4SSG revealed a moderate reduction in HRex (SE = −0.32; *p* = 0.007) and a large increase in HRR (SE = 1.27; *p* < 0.001) from pre-to-post phase 2. Field-based HA can induce physiological changes beneficial to soccer performance in temperate and hot conditions in elite females, and the submaximal running test appears to show HRex responses induced by HA up to 2 weeks following heat exposure.

## Introduction

Athletes are often required to compete in hot and humid environmental conditions, which, if sufficiently hot and coupled with high exercise intensities, can lead to heat stress and, eventually, hyperthermia. Hyperthermia is characterized by an elevation in core (*T*_c_) and skin temperature; an increase in sub-maximal exercise heart rate (HR); and with subsequent dehydration, a reduction in peripheral blood flow and an eventual reduction in sweat rate, resulting in a decline in performance (Chalmers et al., 2014; Racinais et al., 2015; Pryor et al., 2019). Soccer is a sport with high physiological intensities that has the potential to result in greater heat-induced metabolic stress compared to other team sports (Chalmers et al., 2014; Datson et al., 2014). Previous research in high-level soccer players has shown how playing in temperature above 21°C and associated hyperthermia (Buchheit et al., 2011; Carling et al., 2011; Mohr et al., 2012; Mara et al., 2015; Trewin et al., 2018) are negatively correlated to match performance in terms of decreasing the total running distance covered, reductions in high-intensity running speed, and the number of fast directional movements (acceleration and deceleration). Therefore, enhancing environmental preparation is required to optimize soccer performance in the heat.

Repeated training exposures in a hot environment have the potential to induce positive physiological adaptations that can attenuate the negative effects of heat stress by regulating cardiovascular strain while enhancing thermoregulation [e.g., increased plasma volumes (PV) and sweat rates] (Racinais et al., 2015). While isothermically controlled, lab-based heat acclimation protocols have demonstrated improvements in both team and endurance sport performance (Pethick et al., 2018; Benjamin et al., 2019), the resources required to execute an effective acclimation protocol can be expensive (core temperature monitoring), involve extensive equipment (heat chamber), and usually involve non-soccer-specific training (typically cycling) (Buchheit et al., 2011; Racinais et al., 2012; Chalmers et al., 2014). Therefore, the implementation of a field-based, sport-specific heat acclimatization protocol is more ecologically valid and practical for soccer players, while also minimizing time away from training often required for lab-based protocols. Indeed, field-based protocols, such as those utilized by Buchheit et al. (2011, 2013, 2016) and Racinais et al. (2012) previously in male soccer players, have highlighted the potential of repeated heat training exposures to elicit adaptations and offset the impedance of cardiovascular strain during exercise in the heat. This type of training also has the potential to improve sport performance in more temperate conditions (~14–20°C) (Lorenzo et al., 2010; Corbett et al., 2014; Buchheit et al., 2016). Specifically, an enhanced sweat and skin blood flow response, as well as plasma volume (PV) expansion, provides greater cardiac output contributing to the ergogenic response (Lorenzo et al., 2010). This improved cardiac efficiency post-heat acclimatization was evident by reductions in heart rate (HR) during a submaximal running test (Buchheit et al., 2013, 2016). Therefore, it is important to understand how to utilize an effective field-based heat acclimatization protocol in order to preserve, or enhance, soccer performance in both extreme temperatures, as well as during more temperate conditions (Corbett et al., 2014).

While heat acclimatization (via natural environment) has proven to be effective to prepare male soccer players to perform in the heat (Buchheit et al., 2011, 2013, 2016; Mohr et al., 2012; Racinais et al., 2012), other than in female recreational team sport (Sunderland et al., 2008), there has been no research investigating this type of intervention with elite female players. Therefore, the primary purpose of this study was to investigate the response to a natural heat acclimatization camp in a top 10 globally ranked women's national team ahead of the Women's World Cup. This study implemented extensive training load and heat monitoring, as well as various blood measures and practical field-based performance tests.

## Materials and Methods

### Athletes and Experimental Design

Nineteen international-level female soccer players (age: 27.0 ± 5.0 years, body mass: 65.7 ± 5.3 kg, height: 170 ± 6.0 cm, V°O_2Max_: 53.1 ± 3.1 ml kg^−1^ min^−1^) participated in the heat acclimatization (HA) protocol. Three players missed at least one session (injury/scheduling) and were excluded from the final analysis. The study was approved by the University of British Columbia Clinical Ethics Board and conformed to the Declaration of Helsinki. All athletes provided their written consent to participate in all training, monitoring, and testing protocols.

**Figure 1:**
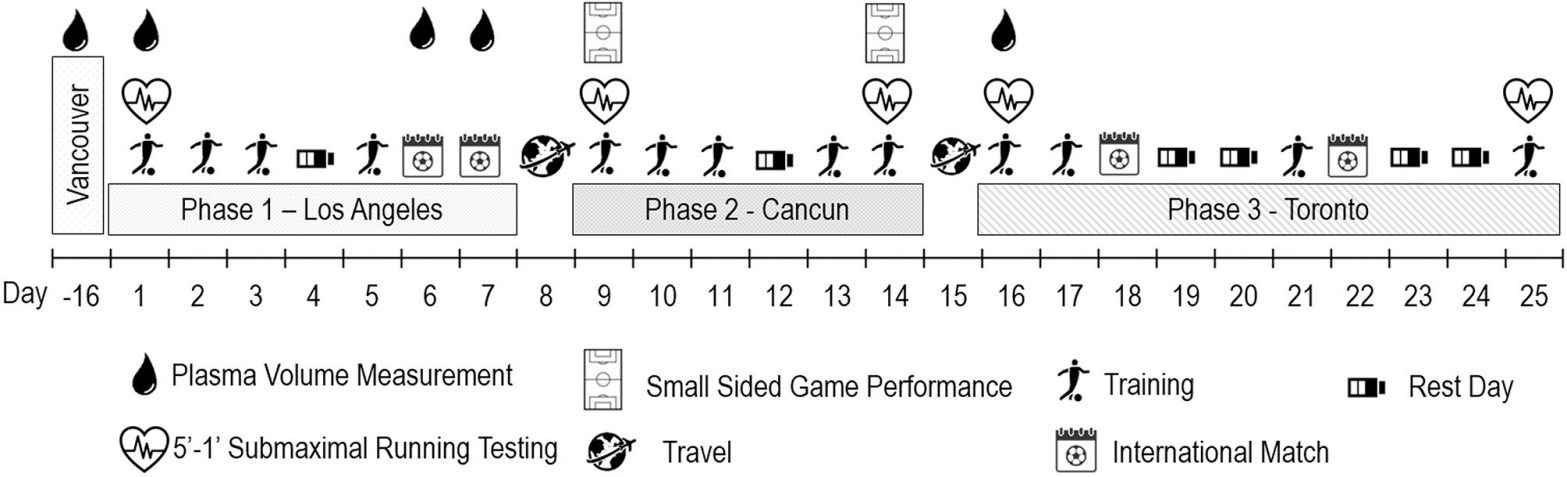
Overview of the study design. Between day−16 and the start of the study, players return home in different places in Canada for a regeneration phase and remote training.

Players were not heat adapted prior to initial testing in April in Vancouver, Canada [mean *T*_ambient_ ~13°C, 72% relative humidity (RH)]. Figure 1 highlights the full testing intervention, training, match, and rest days, over the 25-day period, including the 14-day HA period:

- Phase 1: 8 days in Los Angeles (22.1 ± 3.3°C; 45 ± 9% RH)
- Phase 2: 6 days in Cancun (34.5 ± 1.2°C; 53 ± 4% RH)
- Phase 3: 11 days in Toronto (18.2 ± 4.6°C; 51 ± 20% RH).

### Hematological Parameter Assessments

The training program was adapted, and the athletes were asked to refrain from strenuous exercise and alcoholic and caffeinated drinks in the 12 h prior and to be well-hydrated on all testing days. Body mass was assessed upon arrival; after which, athletes rested in a seated position for 15 min prior to antecubital vein blood sampling. On the first testing occasion (baseline day−16), the optimized carbon monoxide rebreathing protocol was used to determine all hematological parameters (Schmidt and Prommer, 2005) (measured via blood gas analyzer: Radiometer ABL80 FLEX CO-OX analyzer, Denmark), as previously described by our laboratory (Pethick et al., 2019). The Dill and Costill equation (Dill and Costill, 1974) was then subsequently used to determine plasma volumes (PV) as a percent change from the absolute baseline measure.

### Training Load and Player Monitoring

Morning urine specific gravity (USG; Atago PAL 10S refractometer) was monitored throughout (with baseline USG *Z*-scores being calculated from 26 samples for each player over 5 months). *T*_c_ was monitored throughout phases 1 and 2 using a VitalSense Telemetric Monitoring System (Mini Mitter, Philips Respironics, Eindhoven, Netherlands) and thermal sensor (Jonah™ Ingestible Core Temperature Capsule). All *T*_c_ data data were recorded for each athlete ~4–6 times (every ~15 min) throughout the training sessions. Verbal *T*_c_ feedback was provided to the players and coaches in phase, 1 but there was no specific target *T*_c_. In phase 2, *T*_c_ feedback was provided to players and coaches with the goal to maintain a targeted *T*_c_ threshold (i.e., 38.5°C) during training sessions, as generally ~38.5°C appears optimal for HA (Racinais et al., 2015; Periard et al., 2016; Pethick et al., 2018, 2019; Pryor et al., 2019). Area under the *T*_c_ curve or total heat load (AUC) and average Δ*T*_c_ were calculated as previously described by our laboratory (Pethick et al., 2019).

**Figure 2:**
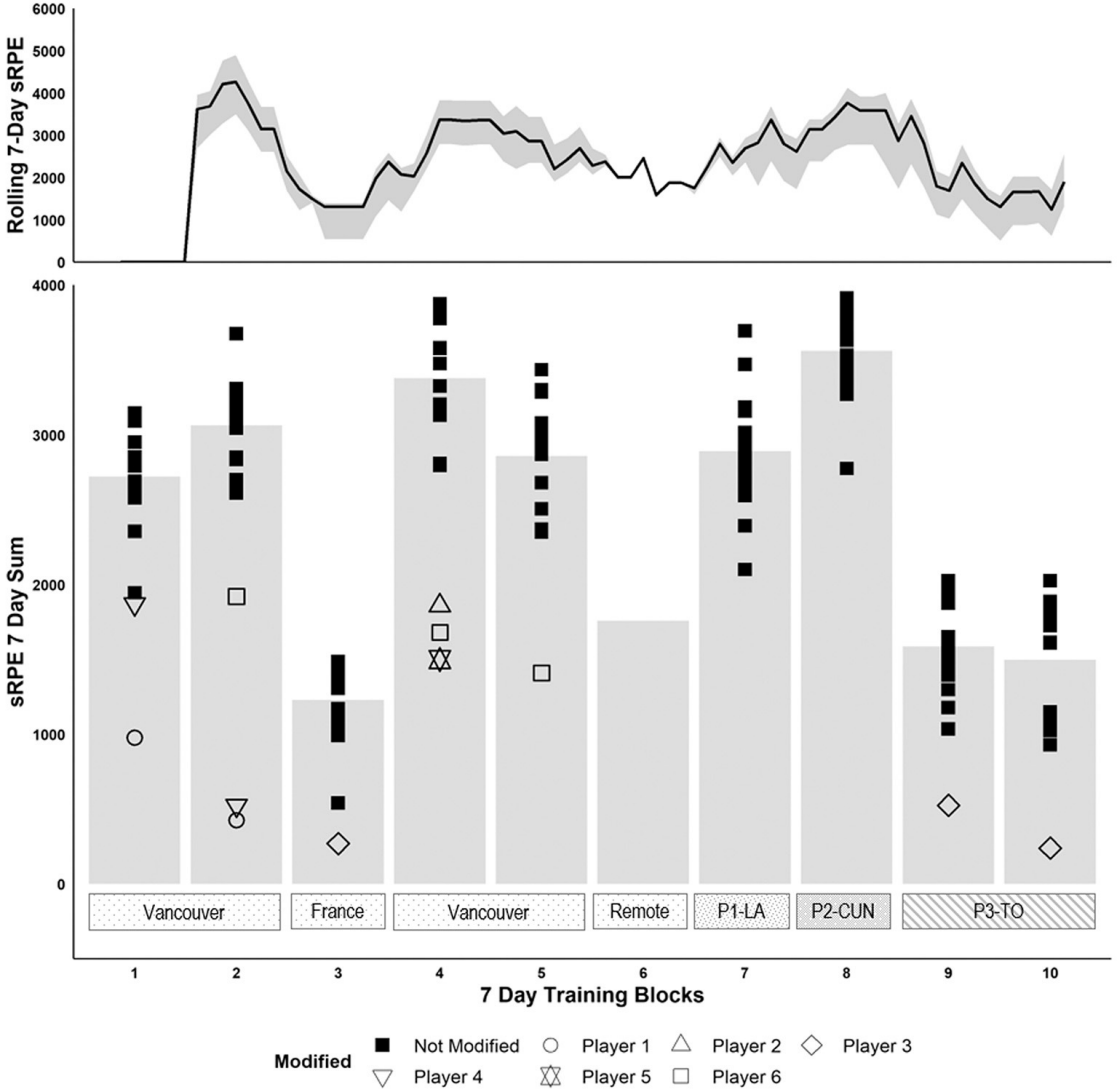
Seven-day RPE rolling sum average (top) and RPE 7-day sum (bottom) across the 10-week world cup preparation. The shaded area in the top graph represents the maximum and minimum for the rolling 7-day sum average. Heat acclimatization occurred during phase 1 in Los Angeles (P1—LA) and phase 2 in Cancun (P2—CUN). Phase 3 was in Toronto in moderate climate (P3—TO).

HR responses were gathered using a Polar Team2 System (1.4.1, Polar Electro Oy, Kempele, Finland), while ratings of perceived exertion (RPE; Borg Scale 1–10) were collected immediately post-session. Session RPE (sRPE) was calculated as a measure of session duration (minutes) multiplied by the RPE value (1–10), as validated for soccer (Impellizzeri et al., 2004). Ratings of thermal comfort (TC: scale of 1–5) and thermal sensation (TS: scale 0–9) were also collected (Young et al., 1987). Players wore a GPS unit incorporating a 100-Hz triaxial accelerometer (Catapult, MinimaxX S4 and Sprint 5.1 software, Australia) to record external training load (Varley et al., 2012). Player movement was categorized as total distance, and any efforts at speeds >16.5 km h^−1^ were recorded as high-speed efforts (Meylan et al., 2017). High Inertial Movement Analysis (High IMA) was categorized as accelerations, decelerations, or changes of direction that exceeded the threshold of 2.5 m s^−2^ (Meylan et al., 2017). As heat-induced blood volume expansion response can also be impacted by training load (Garvican-Lewis et al., 2014), sRPE was also recorded for 5 weeks prior to the HA intervention to establish normal training loads (Figure 2). However, between this 5-week block and the start of the HA (phase 1), players had a 12-day recovery phase, including 10 days at home, where they completed prescribed workouts. Actual training load was not recorded during that time, but sRPE was estimated based on historical responses to the session prescribed. Reported compliance of athletes while training at home was >95% of prescribed.

### The 5-Min Running/1-Min Recovery Submaximal Running Test

A 5-min running/1-min recovery (5′-1′) submaximal test was performed during warm-up on five testing occasions (see Figure 1). Players were tested simultaneously at 12 km h^−1^ over a 40-m shuttle (Mohr et al., 2012). Mean exercise HR (HRex) during the last 30 s of the 5-min running period was recorded with HR recovery index (HRR; %) being calculated by taking the absolute difference between the HRex and the HR in the final 5 s of the recovery period as a percent of HRex. The reliability of the 5′-1′ submaximal running test was assessed on 13 members of the women's national team prior to the HA period on the same athletes across five occasions. Typical error was 2.5 bpm for HRex [confidence limits (CL): 2.03, 3.29], 8% for HRR (CL: 7, 10%), and 0.44 AU for RPE (CL: 0.37, 0.56).

### Small-Sided Game Assessment

A total of four 2-min four-aside small-sided soccer games (4V4SSG) were performed on a reduced pitch (40 × 35 m) with a 2-min rest between games immediately following warm-up on the first (day 9) and last training day (day 14) of phase 2 (Figure 1). Athletes were familiarized with the 4v4SSG prior to the camps. HRex in the small-sided soccer game (SSG) was calculated as the average HR during each of the four SSG (excluding the 2-min rest period). HRR in the SSG was calculated by taking the absolute difference between (i) average HRex during each SSG and (ii) average HR 30 s prior to the next SSG during the 2-min recovery. This value was then divided by the average HRex from each SSG and multiplied by 100 to be expressed as a percentage. The total high IMA and distance were normalized to the 2-min SSG duration and averaged from game one to four to obtain an average game intensity (i.e., meters per minute and high IMA per minute).

### Statistical Analysis

The effect of the HA was analyzed using one-way repeated measures analysis of variance (ANOVA) for the repeated metrics (absolute PV, submax HRR, HRex, RPE, and *T*_c_ pre-training) in R (version 3.4.2, Vienna, Austria). The Tukey honestly significant difference (HSD) *post hoc* procedure was used to control for type I error in making multiple comparisons, to identify the significance difference between conditions. The investigation of residual plots showed a random scatter of points, and the normality plots showed that the residuals fall on a straight line, indicating the normality assumption as appropriate for all metrics. Paired sample *T*-test was conducted with all SSG metrics as well as training variables between phases 1 and 2. The probability of a null effect was set at an alpha level of 0.05.

The effect of the intervention was also provided as standardized effects (Hopkins et al., 2009). Uncertainty in the estimates of effects on laboratory and performance metrics was expressed as 90% confidence limits. Threshold values for assessing magnitudes of standardized effects (changes as a fraction or multiple of baseline SD) were 0.20, 0.60, 1.20, and 2.00 for small, moderate, large, and very large, respectively (Hopkins et al., 2009). These probabilities are not presented quantitatively but were used to make a qualitative probabilistic clinical inference about the effect in preference to a statistical inference based on a null hypothesis test (Hopkins et al., 2009). The effect was deemed unclear when the chance of benefit (a standardized improvement in performance of >0.20) was sufficiently high to warrant use of the intervention, but the risk of impairment was unacceptable (Hopkins et al., 2009). Such unclear effects were identified as those with an odds ratio of benefit to impairment of <66, a ratio that corresponds to an effect that is borderline possibly beneficial (25.0% chance of benefit) and borderline most unlikely detrimental (0.5% risk of harm) (Hopkins et al., 2009).

## Results

### Training Load

**Figure 3:**
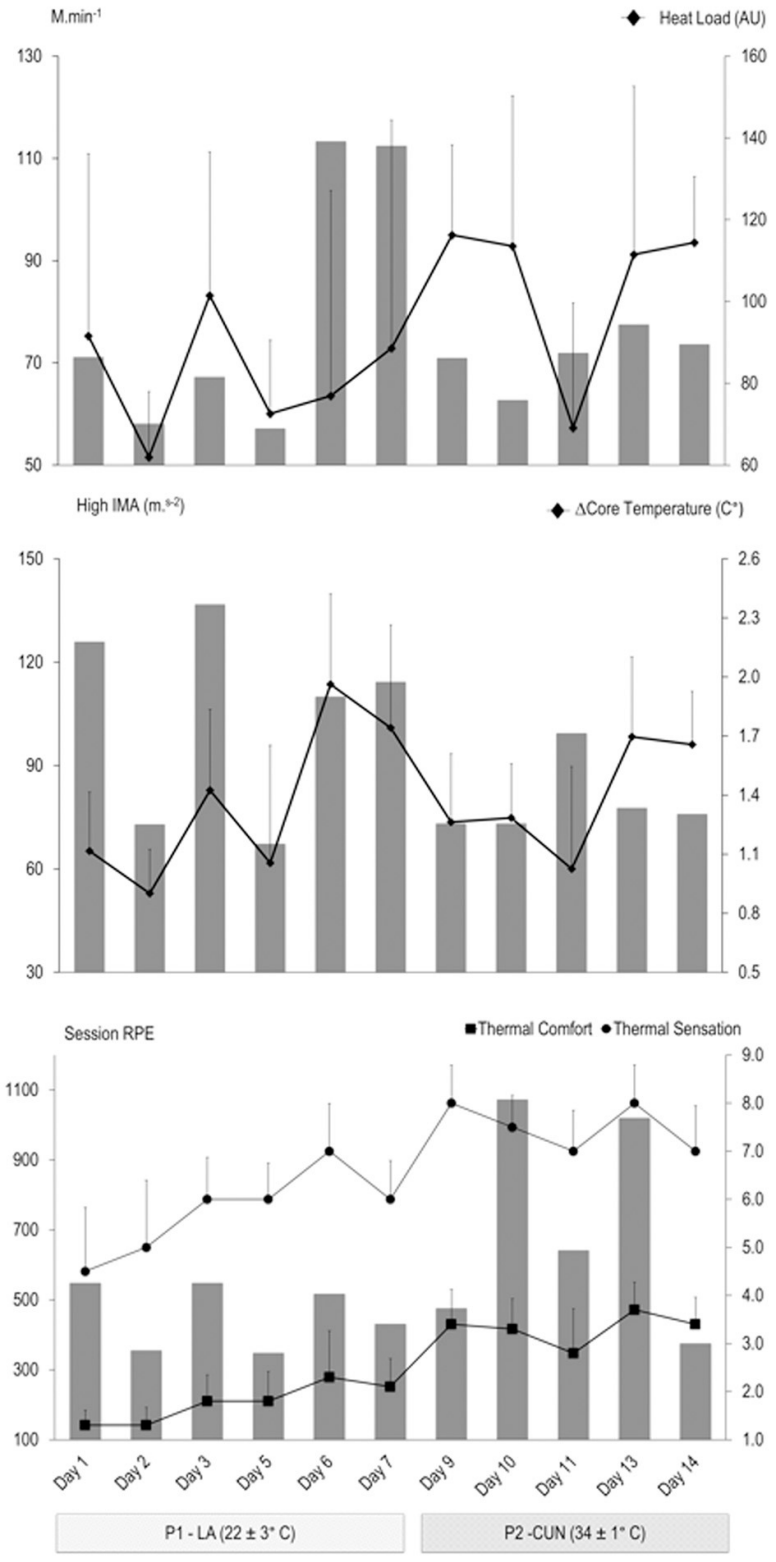
Daily variations in training load and heat stress response during phase 1 in Los Angeles (P1—Los Angeles) and phase 2 in Cancun (P2—Cancun). There was no training on days 4, 8, and 12. Days 6 and 7 were international friendlies.

Data in text and figures are presented as means (±SD) with 90% confidence limits (CL). Figure 2 illustrates the 7-day rolling sum and 7-day weekly block sum of sRPE across 5 weeks prior to, during, and after the HA phases. The peak in 7-day moving average occurred during week 2 of training (4,202 ± 408 AU) and was nearly equivalent to the peak during the HA phase 2 (week 7: 3,630 ± 380 AU). Weeks 3, 9, and 10 were tapering weeks toward international matches and the World Cup. Week 6 was the remote training and sRPE weekly sum was estimated at 1,760 AU, which can be considered a de-loading/recovery week. Table 1 and Figure 3 outline the mean and daily variation in training for the phases 1 and 2. There was a small-to-moderate decrease in external load (e.g., total distance) from phase 1 to phase 2 (*p* < 0.05); however, there was a small-to-very large increase in internal load and thermoregulation from phase 1 to phase 2 (*p* < 0.05) (Table 1). It is important to denote that on days 6 and 7, international games were played, during which meters per minute were much higher because there were very limited stoppages compared to training sessions and players were disregarded if they were subbed out. However, in training sessions, all players were considered and training intensity varied based on coaching within a drill.

**Table 1:** Descriptive (mean ± SD) and standardized differences in weather conditions, thermoregulation responses, and training load for the two phases of heat acclimatization.

	**Phase 1—Los Angeles days 1–7**	**Phase 2—Cancun days 9–14**	**Standardized difference (90% ± CL)**	***P*-value**
Dry bulb temperature (°C)	22.1 ± 3.3	34.5 ± 1.2		
Wind speed (km/h)	2.8 ± 1.4	3.5 ± 0.9		
Relative humidity (%)	47.0 ± 13.0	53.2 ± 4.7		
**Average across sessions**				
Session end *T*_c_ (°C)	38.5 ± 0.2	38.6 ± 0.2^†^	0.59 ± 0.46^*^	0.040
Session Δ*T*_c_ (°C)	1.2 ± 0.3	1.5 ± 0.2^†^	0.80 ± 0.48^**^	0.007
Time Δ*T*_c_ > 1°C (min)	33.1 ± 15.6	34.8 ± 13.7	0.19 ± 0.35	0.565
AUC heat load (AU)	82.2 ± 20.1	105.2 ± 22.3^†^	0.96 ± 0.43^**^	0.001
Thermal comfort (AU)	1.6 ± 0.4	3.3 ± 0.5^†^	2.25 ± 0.33^****^	<0.001
Thermal sensation (AU)	5.5 ± 0.9	7.5 ± 0.6^†^	1.71 ± 0.30^***^	<0.001
RPE (AU)	5.2 ± 0.6	7.0 ± 0.5^†^	2.21 ± 0.31^****^	<0.001
Meters per minute	76 ± 7	71 ± 4^†^	−0.64 ± 0.41^**^	0.013
**Sum across sessions**				
Training duration (min)	492 ± 35	512 ± 21	0.53 ± 0.47^*^	0.069
Session RPE (AU)	2,407 ± 310	3,561 ± 293^†^	2.31 ± 0.40^****^	<0.001
80–89% HRmax (min)	93 ± 22	115 ± 15^†^	0.88 ± 0.39^**^	<0.001
90–100% HRmax (min)	61 ± 25	118 ± 17^†^	1.13 ± 0.39^**^	<0.001
Total distance (km)	31.3 ± 3.5	24.8 ± 1.9^†^	−1.80 ± 0.42^***^	<0.001
High-intensity efforts (count)	174 ± 43	145 ± 30^†^	−0.65 ± 0.27^**^	<0.001
High IMA (m s^−2^)	557 ± 110	408 ± 95^†^	−1.47 ± 0.38^***^	<0.001

*T_c_, core temperature; AUC, area under the curve; HRmax, maximal heart rate; AU, arbitrary units; high-intensity efforts: >16.5 km h^−1^; IMA, inertial movement analysis; high IMA, >2.5 m s^−2^. Standarized effect:*

* *small;*

** *moderate;*

*** *large;*

**** *very large*.

† *Significant difference between phase 1 and phase 2*.

### Plasma Volume

**Figure 4:**
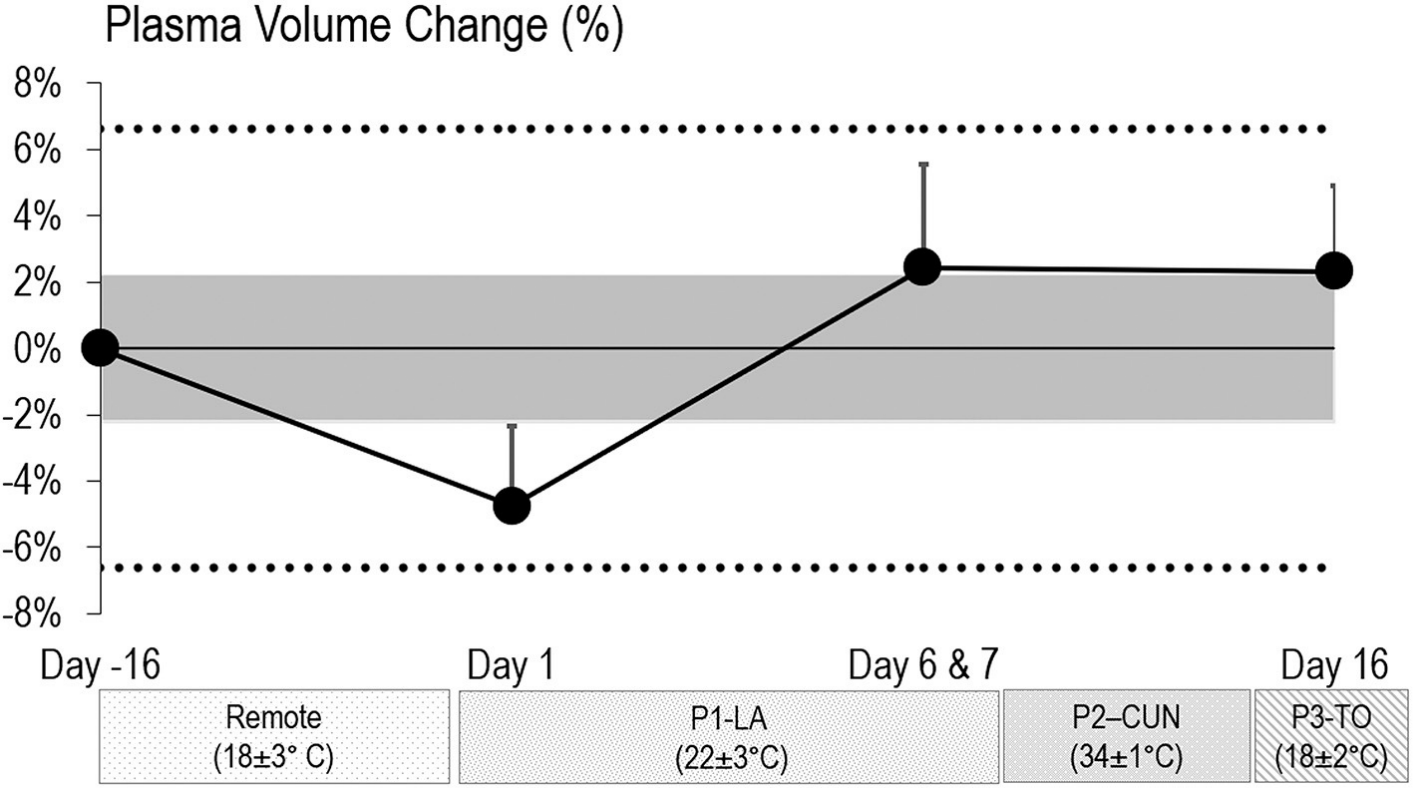
Plasma volume change from baseline testing (day−16) to each follow-up test occasion on day 1 and day 6/7 in Los Angeles (P1—LA) and day 16 in Toronto (P3—TO). No blood draw occurred during phase 2 in Cancun (P2—CUN). The shaded area represents trivial changes, and the dash line represents the lower limit for moderate changes [<0.20, >0.60, and >1.20 of baseline between-subject standard deviation (SD)]. Bars are SDs of changes from baseline to day 6/7 and baseline to day 16. None of the changes were significant (*p* > 0.05).

The mean team USG pre-HA on baseline PV testing (day−16) was 1.020 ± 0.005 (team *z*-score: +1.5 ± 2.0). The mean USG on PV testing on day 1 and day 6 or 7 were 1.014 ± 0.005 (mean *z*-score: +0.1 ± 1.2) and 1.011 ± 0.005 (mean *z*-score: −0.2 ± 0.7), respectively. The mean USG post-HA (day 16) was 1.013 ± 0.005 (mean *z*-score: +0.1 ± 0.8). Figure 4 outlines the change in PV from the absolute average baseline measure via CO re-breathing methodology at day−16 in Canada [PV: 3,935 ± 440 ml (63.5 ± 4.9 ml/kg); BV: 6,561 ± 663 ml; HBmass: 774 ± 88 g (11.8 ± 1.3 g/kg)]. Absolute (*p* = 0.351) and relative (*p* = 0.130) PV changes were non-significant (*F* value = 1.114 and 1.965, respectively). However, there was a small decrease in PV from day−16 (baseline) to day 1 (phase 1 start) (SE = −0.43; CL: −0.66, 0.20). From day 1 to days 6 and 7 (end of phase 1), there was a large increase in PV (SE = 0.64; CL: 0.99, 0.29). From day 1 to day 16 (end of phase 2), there was a large positive increase in PV (SE = 0.63; CL: 0.34, 0.93), and this expansion occurred primarily in phase 1, as the change in PV from days 6 and 7 to day 16 was trivial.

### Core Temperature

Changes in resting *T*_c_ at the start of training were used to further quantify thermoregulation adaptations during the HA phase (*F* value = 17.97). Resting *T*_c_ in day 1 was 37.5 ± 0.18 in Los Angeles and dropped by 0.47 ± 0.13°C (SE = −2.45; −1.24 ± 0.34%; CL: −3.11, −1.78; *p* < 0.001) by day 14 in Cancun. Over the 5 days in Cancun, resting *T*_c_ dropped by 0.22 ± 0.10°C (SE = −1.13; −0.58 ± 0.25%; CL: −1.64, 0.50; *p* = 0.003).

### Submaximal Exercise Performance

**Figure 5:**
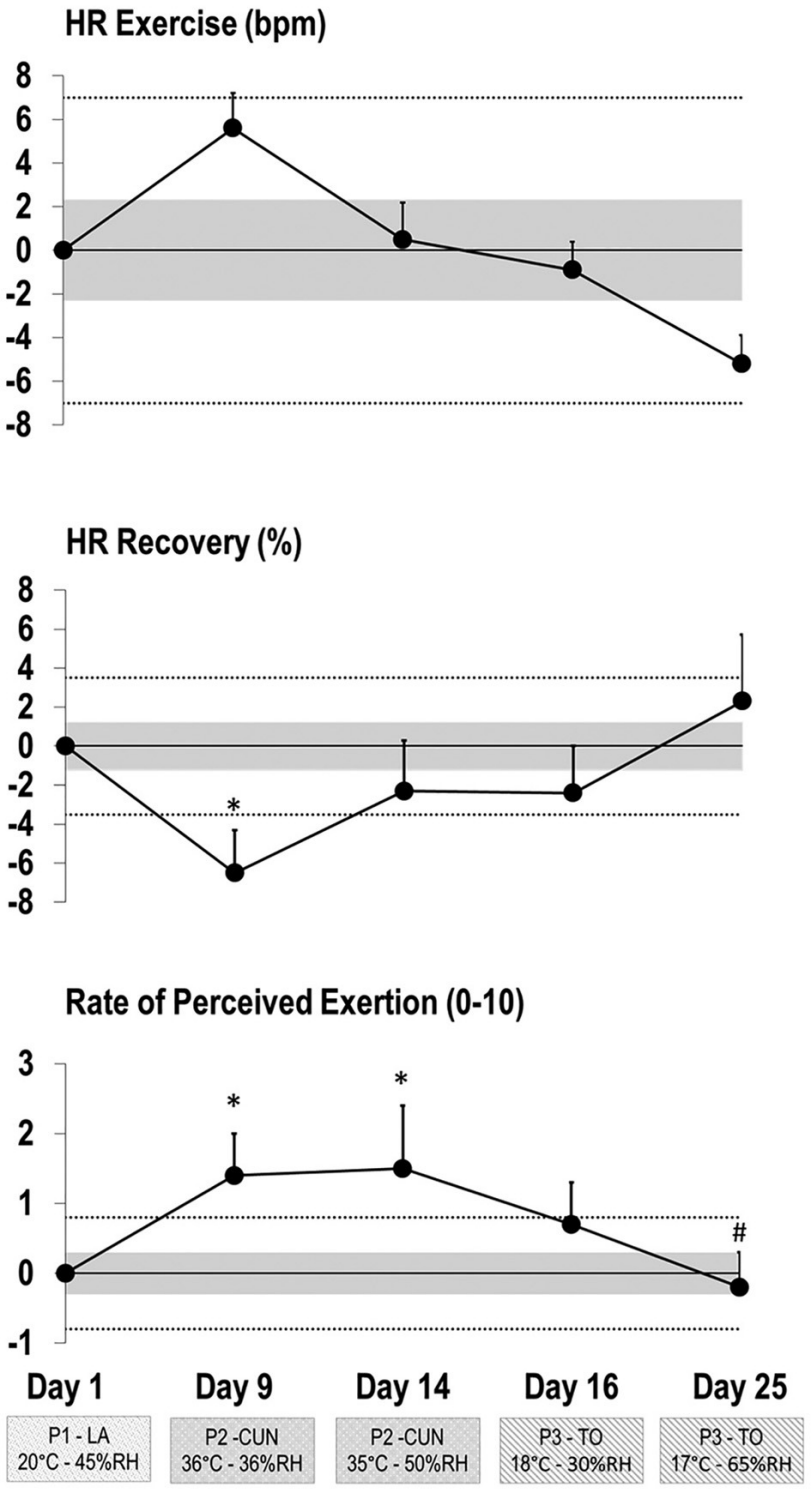
Changes in heart rate (HR) response and rate of perceived exertion (RPE) during the 5′-1′ submaximal running test at each time point compared to day 1 in Los Angeles (P1—LA).The shaded area represents trivial changes, and the dash line represents the lower limit for moderate changes [<0.20, >0.60, and >1.20 of baseline between-subject standard deviation (SD)]. Bars are SDs of changes from baseline to the ensuing testing occasions during phase 2 in Cancun (P2—CUN) and phase 3 in Toronto (P3—TO). *Significant difference from LA (*p* < 0.01); ^#^significant difference from CUN 1 and 2.

Figure 5 outlines the change in 5′-1′ submaximal running performance from day 1 to assess the change in HRex (170 ± 11 bpm), HRR (39 ± 6%), and RPE (3.5 ± 1.3 AU) during HA. HRex did not show any significant changes across the testing occasion (*F* value = 0.95) while HRR and RPE did (*F* value = 2.72 and 7.94, respectively). From day 1 to day 9 (phase 2 start), there was a small increase in HRex (SE = 0.45; CL: 0.32, 0.57; *p* = 0.442), a moderate decrease in HRR (SE = −1.02; CL: −1.38, −0.67; *p* = 0.037), and a moderate increase in RPE (SE = 1.00; CL: 0.53, 1.47; *p* = 0.003). Following five HA sessions over 6 days in phase 2 (days 9 to 14), there were no significant changes in the variables of interest (*p* > 0.05), but the standardized effect indicated a small decrease in HRex (SE = −0.49; CL: −0.67, −0.31) and a small increase in HRR (SE = 0.53; CL: 0.04, 1.02) while RPE remained consistent (AU = 4.7 ± 0.9 to 4.9 ± 0.9). From the end of phase 2 on day 14 to day 16 (start of phase 3), there were no significant changes in the various metrics (*p* > 0.05) and SE analysis only revealed a small decrease in RPE (SE = −0.41; CL: −0.65, −0.18). There was also a moderate increase, yet not significant (*p* = 0.486), in RPE (SE = 0.45; CL: 0.03, 0.88) from day 1 to day 16. From day 1 to day 25 (end of phase 3), even though not significant, there was a moderate decrease in HRex (SE = −0.42; CL: −0.52, −0.3; *p* = 0.442) and a moderate increase in HRR (SE = 0.37; CL: −0.17, 0.92; *p* = 0.999), while RPE was similar to what was observed on day 1 during initial testing (SE = −0.04; CL: −0.41, 0.33; *p* = 0.004).

### Small-Sided Games

**Figure 6:**
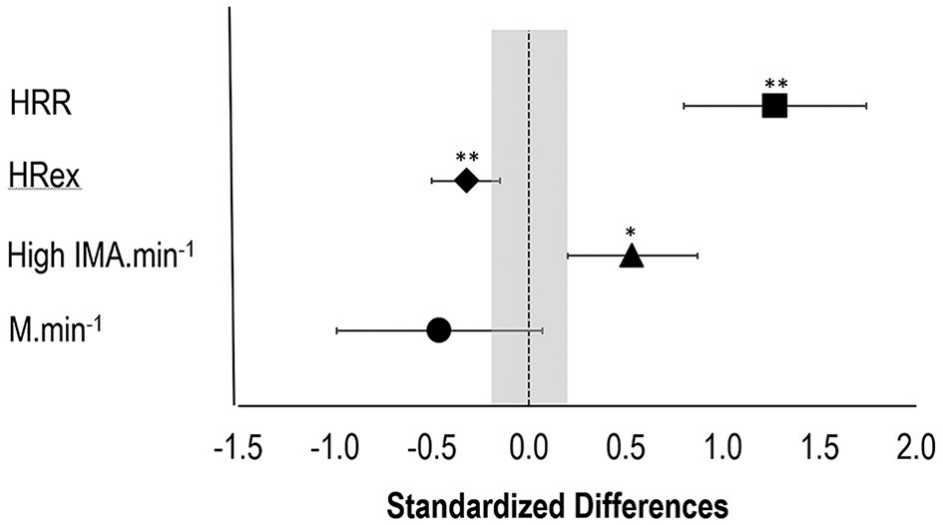
Standardized differences (±90% confidence intervals) in physical performance during a four-a-side small-sided soccer game played in phase 2 in Cancun on day 9 (36°C−36% RH) and the last day of phase 2 on day 14 (35°C−50%RH). The shaded gray area represents trivial differences (i.e., <0.20). *Significant difference (*p* < 0.05 and **p < 0.01) between day 9 and day 14. HRR, HR recovery; HRex, HR at exercise; high IMA min^−1^, number of high inertial movement action (>2.5 m s^−2^) per minute; m min^−1^, meters per minute.

Figure 6 outlines the standardized differences between the 4v4SSG at the start (day 9) and end (day 14) of phase 2. HRex and HRR were both different from days 9 to 14 with HRex decreased (−3.5 bpm; CL: −5.5, −1.6; *p* = 0.007) and HRR increased (5.7 ± 1.6%; CL: 4.1, 7.4; *p* < 0.001). Additionally, there was an increase in high IMA activity (20.1%; CL: 6.6, 35.2; *p* = 0.015) but not in meters per minute (−4.7%; CL: −9.6 to 0.7; *p* = 0.144), although both changes were small.

## Discussion

Our study is the first to investigate the effect of a field-based heat acclimatization protocol on PV, submaximal HR responses (as a marker of cardiovascular adaptations), and core temperature monitoring within soccer-specific, performance-based testing protocols in international female soccer players. The key finding was that a practical real-world HA camp induced relevant improvements in sport-specific performance metrics (Figure 6), although some of these positive adaptive responses were delayed coming out of the HA camp (Figure 5). These changes occurred along with a non-significant trend for PV expansion, which may be associated with high inter-athlete variability.

Previous findings in male soccer players (Buchheit et al., 2011, 2013, 2016; Racinais et al., 2012) show that heat acclimatization can lead to an increase in PV and were corroborated in elite female soccer athletes in the current study, although the PV change failed to reach significance (Figure 4). Laboratory studies utilizing controlled hyperthermia acclimation protocols have reported PV increases of 4–15%, improving cardiovascular stability via maintenance of cardiac output and reductions in HRex, leading to performance enhancement (Chalmers et al., 2014; Periard et al., 2015, 2016; Racinais et al., 2015; Casadio et al., 2017; Benjamin et al., 2019; Pryor et al., 2019). While laboratory HA protocols are more controlled, they are less practical and potentially less effective than field-based HA, which has been shown to induce better peripheral adaptations from sport-specific training and better maintenance of skills (Pryor et al., 2019). Collectively, four field-based HA protocols utilizing team sport players demonstrated a mean PV expansion of 5.4% (Buchheit et al., 2011, 2013, 2016; Racinais et al., 2012), similar to the current study's trend of 7.4 ± 3.6% across phase 1 (LA) and phase 2 (Cancun; Figure 4). This would be a similar PV expansion to what we found during an indoor heat acclimation study in some of the same athletes, where we demonstrated ~9% PV increase (Pethick et al., 2018). Additionally, some soccer-specific, field-based HA protocols have previously demonstrated improved sport performance following HA (Sunderland et al., 2008; Buchheit et al., 2011, 2013). In two different studies featuring natural heat acclimatization, Buchheit et al. (2011, 2013) reported increases in PV following both 1 week (7%) and 2 weeks (5.6%) of HA, which translated into substantial improvements in athletic performance with the greater improvement seen with the longer HA protocol (+7 vs. 44% in YOYOIR2, respectively). It should be noted that beyond sex-based differences (male vs. female players in the current study), the significant findings of the prior heat acclimatization studies (Buchheit et al., 2011, 2013, 2016; Racinais et al., 2012) all featured longer durations in high heat (7–14 days) and higher heat stress (up to 43°C) compared to the current study.

The present findings suggest a meaningful 7.4 ± 3.6% increase in PV from the start of phase 1 to the end of day 16 (Cancun; Figure 4). Indeed, this potential shift in PV was probably caused by a combination of increases in training load after the 10 days recovery remote phase and throughout the 7 days of phase 1, as there is evidence that physical training alone induces PV expansion in elite endurance athletes (Garvican-Lewis et al., 2014; Bejder et al., 2017). However, exercise alone is certainly not solely responsible for this PV expansion, as there was significant heat stress throughout phase 1 in LA, as demonstrated by the various elevated *T*_c_ metrics during phase 1 (Table 1) as well as the drop in resting *T*_c_, a key indicator of HA (Periard et al., 2015, 2016; Racinais et al., 2015). Interestingly, despite phase 2 being significantly warmer than phase 1, there did not appear to be further PV expansion, probably due to the fact that the players were already at the peak of their aerobic fitness and had endured a large training load in preparation for the World Cup (Figure 2). Indeed, the athletes' ability to expand PV to an even greater extent may have been limited by a potential ceiling effect, as it has been previously demonstrated that a low baseline PV was a significant predictor of HA-induced increases in PV (Pethick et al., 2019). While further studies are needed to more clearly establish the effects of PV expansion and the ergogenic potential of HA in field-sport/team athletes, even minor physiological improvements are likely to benefit athletic performance at the international level (Malcata and Hopkins, 2014).

The literature has consistently demonstrated improvements in submaximal exercise performance following HA characterized by a reduced HRex and an increased VO_2max_ (Periard et al., 2016). However, the evidence for observed improvements specifically in HR response during submaximal field sport testing in elite athletes is more limited (Buchheit et al., 2011; Racinais et al., 2014, 2015). In line with previous literature in elite male soccer players (Buchheit et al., 2011), our data also demonstrated meaningful improvements in HR response (Figure 5) in international female soccer players during submaximal running pre-post HA; however, this same study found no improvement in HRR (Buchheit et al., 2011). This limited response in HRR was suggested to be the result of using low running speeds during the test (9 km h^−1^ instead of 12 km h^−1^) and by extension lower HRs, for the elite-level players (Buchheit et al., 2011). By contrast, the current study implemented the higher running speed of 12 km h^−1^ and did find an improvement in HRR. Thus, the 5′-1′ submaximal running test used in the current study was effective in tracking both HRR and HRex to monitor HA-induced cardiovascular adaptations throughout the various phases. As outlined in Figure 5, initial submaximal performance during phase 2 (day 9 ~34°C) was more stressful than during the mild conditions in phase 1 (day 1 ~22.1°C). Likewise, Buchheit et al. (2016) observed an increase in RPE during HA (34.9°C) when athletes underwent a 12-°C increase in temperature over 2 days. After six days, players in the current study acclimatized aerobically to the heat as evidenced by the internal response (HR and HRR) to submaximal running by the end of phase 2 (day 14) returning to similar levels as at the start of phase 1 (Figure 5). Therefore, in support of previous findings, both cardiovascular and perceptual adaptation can be observed in as little as five field-based HA sessions (Chalmers et al., 2014; Periard et al., 2016).

A novel finding from the submaximal test was the large improvement in HR response observed 11 days post-HA in a temperate condition (Figure 5; day 25). This finding challenges previous research suggesting that competitive athletes may only retain HA for up to 1 week (Pandolf, 1998); it also highlights the importance of a submaximal retest up to 2 weeks post-HA, since the fitness gains may not be complete for several days post-acclimation and/or athletes needs to shed fatigue from the extra training stress induced by a hot environment (Table 1). This finding is further supported by Buchheit et al. (2016) who utilized a 4-min submaximal test (12 km h^−1^) before, immediately post, and 3 days after a HA camp and found that the greatest reduction in HR response did not occur immediately, but 3 days following the camp. This delayed cardiovascular/aerobic adaptation post-HA could be partly attributed to an improvement in neuromuscular efficiency after recovering from HA (Buchheit et al., 2016). Furthermore, a recent study has shown that intense training in the heat can cause impairments in performance, most probably due to the added environmental stress causing early signs of over-reaching (Reeve et al., 2019). Although external training load was less in phase 2 than phase 1 (Figure 3), internal load (e.g., time above 90% HRmax) was actually greater. Previous evidence has shown increased sympathetic activity and a reduced vagal activity during periods of intensified load (Baumert et al., 2006), which could have affected the HR response to submaximal exercise. These effects are typically a result of over-reaching and are known to resolve after 3–4 days of recovery (Baumert et al., 2006; Buchheit, 2014). Studies have shown that physical fitness typically plateaus at the final stages of in-season training (Clark et al., 2008). This suggests that the markers of improved aerobic capacity in the current study are not exclusively due to a training adaptation, due to the fact that players were at their highest aerobic fitness (2 weeks before the FIFA World Cup), but due to the additional stimulus of HA. The optimal time to engage in HA without having to re-acclimate before a competition is still uncertain and requires further investigation, although some aspects of heat periodization, and retention, are starting to emerge in the literature (Casadio et al., 2016, 2017).

Small-sided soccer games have been validated as an effective test for replicating match play as the smaller field dimensions allow for continued ball contact and short intermittent running in order to attain similar cardiovascular, mechanical, and technical demands as in competitive matches (Dellal et al., 2011; Lacome et al., 2018). Just five training sessions in hot conditions (phase 2) induced meaningful heat adaptation that improved the density of explosive actions and associated HR response and recovery during and between 4v4SSG (Figure 6). The small and non-significant decrease in average intensity (meters per minute) could be a response to the players choosing to be more explosive and to cover less distance at low speed to achieve more ball interactions due to greater comfort and lower HR response in the heat (Dellal et al., 2011; Lacome et al., 2018). Those locomotive changes are unlikely to be a response to coaching as the SSG was set up to be free play and did not have any explicit or implicit coaching cues. By design, 4v4SSG is characterized by a greater density in explosive actions than match play or bigger SSG (Lacome et al., 2018), and it could be argued that an increase in high IMA is more relevant to a positive locomotive change in a 4v4SSG as compared to meters per minute. Still, SSGs provide a valid replication of the performance physical demands of competitive match play under more control conditions (e.g., limited ball out of play, less players, and no set pieces) and allow for a meaningful interpretation of soccer match performance, when an environmental factor such as heat is added to training (Fenner et al., 2016).

With this training camp occurring only 2 weeks prior to the FIFA World Cup, we were unable to include a control group. Therefore, the observed ergogenic effect of HA in the current study may be confounded by the effects of training load, travel fatigue, changes in sleep, recovery, diet, and motivation. Another limitation was the inability to control for the effect of menstrual phase as this has previously been shown to affect the *T*_c_ response in females (Fortney et al., 1994; Logan-Sprenger et al., 2012).

There are also a number of factors that can impact the increase in PV including (i) the acclimatization day when PV is measured; (ii) the type of method used to measure PV; (iii) the hydration state when measured; (iv) *T*_skin_ at the time of measure; and (v) fluctuation in training load (Sawka et al., 1984; Periard et al., 2016). However, beyond environmental strain, training load is also a significant impactor of PV responses, and thus, our study took a lot of care to characterize the training loads throughout the entire study (Table 1). Indeed, there was not a significant difference between phase 1 and phase 2 for total training duration, and phase 2 actually featured significantly less total distance run and high-intensity efforts (Table 1), and we therefore believe most of the outcomes are due to the heat stress, and not training volumes, and associated cardiovascular responses to heat (as shown by higher HR's during phase 2). While there was a positive increase in PV overall, the lack of consistent elevation of *T*_c_ at a target threshold on a daily basis during phase 2 training likely limited the increase in total body water. It is also possible that athlete hydration status within each training session may have impacted the extent of PV expansion, as hydration level can cause fluid shifts between the circulatory system and interstitial spaces during exercise in the heat (Sawka et al., 1984). While we were able to use a consistent sampling site while measuring PV, we were unable to account for the change in fluid intake throughout both phases of acclimatization. Furthermore, Hct and Hb are highly responsive to changes in plasma osmolality; therefore, the inability to effectively monitor plasma osmolality via fluid intake may have resulted in a larger variation of Hct during the training camp, which in turn would have largely impacted the accuracy of blood sampling and the overall change in PV (Kavouras, 2002; Watson and Maughan, 2014). However, the average USG values in athletes on blood testing days were within the norms of adequate hydration status.

There are also several methodological limitations to using the Dill and Costill method as opposed to the CO rebreathing method, which has proven to be more effective in reporting consistent and accurate values of PV (Schmidt and Prommer, 2005; Alis et al., 2015). While it would have been preferential to continue to use the CO rebreathing method beyond baseline testing and within both phases of the training camp, this method requires a longer protocol (~30–40 min per athlete), and we wanted to limit the time taken out of an athlete's schedule prior to competition. Therefore, the CO rebreathing technique was only utilized to obtain an absolute baseline for which the percentage change in PV was determined throughout the remainder of the camp.

## Conclusion

The present research provides an insight on the efficacy of a 2-week field-based HA protocol to elicit physiological adaptations and improved physical performance-related metrics, with ergogenic effects demonstrated in both temperate and hot conditions. The current findings support the use of daily, on-field training load monitoring of GPS external load, HR metrics, *T*_c_ and heat load real-time monitoring, and session RPE to monitor intensity and adaptations within HA. It is also recommended that athletes perform a sport-specific measure of performance, and while the current study utilized a 4v4SSG, it could be more effective to utilize eight players over a larger field dimension (8v8SSG) and to use 6–8-min small-sided games instead of 2-min games. Furthermore, due to potential immediate residual fatigue from heat, monitoring physiological and performance metrics during HA for up to 2 weeks post-HA is recommended. In terms of performance, there is no current evidence of neuromuscular fatigue monitoring in combination with HR response during HA, which could potentially be used as a secondary method to monitor the fatigue induced by environmental heat stress, allowing one to fully understand the periodization puzzle of HA integration in preparation for a major event.

## Data Availability Statement

The original contributions presented in the study are included in the article/supplementary material, further inquiries can be directed to the corresponding author/s.

## Ethics Statement

The studies involving human participants were reviewed and approved by University of British Columbia Clinical Ethics Board. The patients/participants provided their written informed consent to participate in this study.

## Author Contributions

CM, KB, WP, and JT were all involved in the data collection and processing of the data. Data analysis was primarily conducted by CM, KB, TS, and MK. All authors took part in the design of the study and contributed to manuscript write up and final edits.

## Conflict of Interest

The authors declare that the research was conducted in the absence of any commercial or financial relationships that could be construed as a potential conflict of interest.
